# A checklist for reproducibility in electrochemical nitrogen fixation

**DOI:** 10.1038/s41467-022-32146-x

**Published:** 2022-08-11

## Abstract

In this editorial, we present a checklist that aims to improve the data reproducibility in the field of electrochemical N_2_ fixation.

Nitrogen fixation, a process by which inert N_2_ is converted to N-containing compounds, is crucial for sustaining life on Earth. However, this process is extremely challenging due to the high energy penalty in breaking the stable N_2_ triple bond. Currently, N_2_ is commonly activated via the energy-intensive Haber–Bosch process to produce NH_3_. From NH_3_, other N-containing compounds, such as nitric acid and urea, are manufactured via subsequent reactions. Nevertheless, the high energy consumption and carbon emissions of the Haber–Bosch process have spurred researchers to explore greener alternative approaches.

In the past decade, electrochemical N_2_ reduction to ammonia has been considered an appealing alternative to the Haber–Bosch process due to the potential to avoid the high temperature and pressure conditions typical of that reaction, in addition to its compatibility with renewable energy sources. This field has thus attracted intensive research and has expanded to the synthesis of N-containing products beyond NH_3_, including urea production from N_2_ co-reduction with CO_2_ and nitric acid production from N_2_ oxidation.

Despite these tremendous efforts, progress in this field has been heavily impeded by the ambiguity of the nitrogen source for the observed N-containing products. This ambiguity arises from ubiquitous N-containing contaminants in the electrocatalytic system such as NH_3_ and NO_*x*_—rather than just N_2_—that can contribute to the observed N-containing products. The critical role of these N-contaminants has become clearer over time, and some pioneering works (refs. 1–6) have made appreciable efforts toward addressing this issue and highlighting the need for the implementation of strict experimental protocols.

Here, we present our electrochemical nitrogen fixation checklist. The goal of this checklist for the field of electrochemical N_2_ fixation is to ensure a rigorous set of experimental parameters for data acquisition, to improve the data reproducibility between laboratories, and to promote robust and sustainable scientific growth.

“The goal of this checklist for the field of electrochemical N_2_ fixation is to ensure a rigorous set of experimental parameters for data acquisition, to improve the data reproducibility between laboratories, and, to promote robust and sustainable scientific growth.”

A full response to this checklist is required for every manuscript submitted to *Nature Communications* on this topic. This response letter will be evaluated by the editorial team and reviewers.
